# Adverse effects of small-volume red blood cell transfusions in the neonatal population

**DOI:** 10.1186/2046-4053-3-92

**Published:** 2014-08-20

**Authors:** Amy Keir, Sanchita Pal, Marialena Trivella, Lani Lieberman, Jeannie Callum, Nadine Shehata, Simon Stanworth

**Affiliations:** 1 School of Paediatrics and Reproductive Health, University of Adelaide, Adelaide 5005, Australia; 2 Department of Neonatal Medicine, Level 1 Queen Victoria Building, Women's and Children's Hospital, 72 King William Road, North Adelaide, South Australia 5006, Australia; 3 Rosie Neonatal Unit, Cambridge University Hospitals NHS Foundation Trust Cambridge, Cambridge CB2 0QQ, UK; 4 Centre for Statistics in Medicine, University of Oxford, Oxford OX2 6UD, UK; 5 Transfusion Medicine, University Health Network, Toronto M5G 2N2, Canada; 6 Department of Clinical Pathology, University of Toronto, Toronto M5G 2M9, Canada; 7 Transfusion Medicine and Tissue Banks, Sunnybrook Health Sciences Centre, Toronto M4N 3M5, Canada; 8 Department of Laboratory Medicine and Pathobiology, University of Toronto, Toronto M5S 1A1, Canada; 9 Department of Medicine, Mount Sinai Hospital, Toronto M5G 1X5, Canada; 10 National Health Service Blood and Transplant/Oxford University Hospitals NHS Trust, Oxford OX3 9DU, UK

**Keywords:** Transfusion/adverse effects, Neonates, Systematic review, Red blood cell transfusion, Transfusion reaction

## Abstract

**Background:**

Adverse transfusion reactions in the neonatal population are poorly understood and defined. The incidence and pattern of adverse effects due to red blood cell (RBC) transfusion are not well known, and there has been no systematic review of published adverse events. RBC transfusions continue to be linked to the development of morbidities unique to neonates, including chronic lung disease, retinopathy of prematurity, intraventricular haemorrhage and necrotising enterocolitis. Uncertainties about the exact nature of risks alongside benefits of RBC transfusion may contribute to evidence of widespread variation in neonatal RBC transfusion practice.

Our review aims to describe clinical adverse effects attributed to small-volume (10–20 mL/kg) RBC transfusions and, where possible, their incidence rates in the neonatal population through the systematic identification of all relevant studies.

**Methods:**

A comprehensive search of the following bibliographic databases will be performed: MEDLINE (PubMed/OVID which includes the Cochrane Library) and EMBASE (OVID). The intervention of interest is small-volume (10–20 mL/kg) RBC transfusions in the neonatal population.

We will undertake a narrative synthesis of the evidence. If clinical similarity and data quantity and quality permit, we will also carry out meta-analyses on the listed outcomes.

**Discussion:**

This systematic review will identify and synthesise the reported adverse effects and associations of RBC transfusions in the neonatal population. We believe that this systematic review is timely and will make a valuable contribution to highlight an existing research gap.

**Trial Registration:**

PROSPERO, CRD42013005107

http://www.crd.york.ac.uk/PROSPERO/display_record.asp?ID=CRD42013005107

## Background

Anaemia of prematurity (AOP) is a multifactorial condition with diminished plasma erythropoietin (EPO) levels in response to anaemia and hypoxia, reduced red cell life span, phlebotomy losses for laboratory testing, limited transplacental transfer of iron due to premature birth and dependence on hepatic EPO production [1]. Small-volume red blood cell (RBC) transfusions are often used to manage AOP with over 90% of preterm neonates with a birthweight at <1,000 g receiving at least one RBC transfusion [2,3]. RBC transfusions are given with the assumption that the transfusion will lead to an increase in oxygen delivery to tissues, thereby providing a rapid and effective intervention.

However, RBC transfusions are biological products, with recognised risks. Adverse effects may be classified broadly as those related to errors in the processing, storage and administration or as actual medical complications. Interpretation of the data from the UK Serious Hazards of Transfusion (SHOT) National Haemovigilance Scheme of a population-based epidemiological study of transfused patients has suggested that a disproportionate increased number of adverse events occur in children compared to adults, and more so in neonates [4]. A significant proportion of these reports were related to transfusion errors, including transfusion of an incorrect blood component. While SHOT has received numerous reports related to transfusion errors in the neonatal age group, there have been relatively fewer adverse reactions to transfusion reported. In the 2011 Annual SHOT report [5], there were no reports of transfusion-related lung injury (TRALI) in neonates. There were five paediatric reports classified as transfusion-associated circulatory overload (TACO) that included one neonate. It seems likely that there is under-recognition and/or under-reporting of transfusion-related adverse events in neonates [6,7] due to pre-existing critical illness, in particular around the recognition of TRALI [8] as many preterm neonates having intercurrent respiratory disease. This is compounded by the difficulties in defining adverse transfusion events in a neonatal setting.

There are several recognised potential adverse associations related to RBC transfusions unique to neonates [9]. Associations between receipt of RBC transfusions and development of necrotising enterocolitis [10], intraventricular haemorrhage [11,12] retinopathy of prematurity [13], chronic lung disease [14] as well as mortality [15,16] have all been described. The exact nature of these potential risks, alongside benefits of RBC transfusions, has likely contributed to widespread variation in neonatal RBC transfusion practice [17]. To date, there has been no systematic collation of adverse effects due to, or associated with, RBC transfusion in neonates nor assessment of the degree to which biases operate to mitigate for or against the strengths of associations with risks.

Our review aims to describe clinical adverse effects attributed to small-volume (10–20 mL/kg) RBC transfusions and, where possible, their incidence rates in the neonatal population through the systematic identification of all relevant studies. It is likely that our review will find that reporting of adverse events related to neonatal transfusion is variably described in the literature and there is a need for standardisation of definitions in this area.

## Methods/design

This review will be reported in accordance with the Preferred Reporting Items for Systematic Reviews and Meta-Analyses (PRISMA) statement [18]. It has also been registered in the PROSPERO international prospective register of systematic reviews (registration number: CRD42013005107).

### Study eligibility

We will include both randomised (including cluster-randomised and quasi-randomised) and non-randomised studies (including observational, cross-sectional, experimental and retrospective), with the *proviso* that any analysis will be carried out separately for randomised and non-randomised studies. Only studies examining the effects of RBC transfusion on neonates and have at least one outcome deemed relevant to our review will be included. Studies will not need to have a comparator group to be included; however, only those with a comparator group will be used in any meta-analysis. Our review will also focus its interpretation on those studies with a comparator group.

We will exclude reviews, case series with less than five neonatal participants, case reports, animal studies and laboratory (in vitro) studies. We will exclude studies that examine exchange transfusion, foetal (in utero) transfusion, large-volume transfusions and transfusions used in cardiac surgery and for extracorporeal membrane oxygenation (ECMO). These studies were excluded as we have chosen to focus on the potential adverse effects of small-volume RBC transfusions only.

### Population

Neonates who received at least one RBC transfusion will be considered. Infants are defined for the purposes of this review as neonates less than 28 days of age and premature neonates (<37 weeks gestation) up to four weeks post-term corrected age.

### Interventions

The intervention of interest is small-volume (10–20 mL/kg) RBC transfusions.

### Comparators

For studies with a comparator group, we will include studies comparing

1. RBC transfusion with no RBC transfusion

2. Higher versus lower RBC transfusion threshold (or comparisons among RBC transfusion thresholds)

3. Higher versus lower RBC transfusion volumes

4. RBC transfusion products (e.g. leukodepletion, irradiation, age of RBC product, anticoagulant preparation versus non-modified)

5. RBC transfusion with an alternative therapy (e.g. erythropoietin-stimulating agents)

### Outcomes

Depending on data availability, our outcomes will be considered separately for ‘strong’ (e.g. immune-mediated transfusion reactions) and ‘less certain’ (e.g. late-onset sepsis, NEC, BPD, severe ROP, etc.) causal pathways from transfusion to event.

#### Primary outcomes

1. Mortality associated with receipt of RBC transfusion

i. Within 24–48 h of receipt of a RBC transfusion.

ii. Before discharge from initial hospitalisation.

2. Complications during hospital stay

Chronic lung disease (defined as requirement of supplemental oxygen at 36 weeks gestation), retinopathy of prematurity (grade 3 or above) [19], necrotising enterocolitis (stage 2 or greater using Bell's criteria) [20], intraventricular haemorrhage (grade 3 or 4) [21], adverse neurodevelopmental outcomes (at 18–24 months corrected age), cerebral palsy diagnosed following physician assessment or developmental delay (IQ or DQ > 2 standard deviations below the mean on a validated assessment tool of cognitive function), or blindness (visual acuity).

#### Secondary outcomes

1. Adverse transfusion events

Immune-mediated transfusion reactions (acute haemolytic transfusion reactions, febrile non-haemolytic transfusion reactions and transfusion-related acute lung injury) within 48 h of receipt of RBC transfusion.

Acute non-immune-mediated transfusion reactions (transfusion-related circulatory overload, metabolic complications including hypocalcaemia, hyperkalaemia, hyper/hypoglycaemia and hypothermia) within 48 h of receipt of RBC transfusion.

Alloimmunisation, transfusion-associated graft versus host disease, post-transfusion purpura, infectious adverse effects (transfusion-transmitted infection, e.g. hepatitis B, hepatitis C, HIV, HTLV, parasites), bacterial contamination/sepsis, incorrect blood component transfused and/or adverse events or reactions associated with directed donation.

If data availability allows, we will examine adverse transfusion events in the individual categories as outlined above.

2. Longer-term outcomes

Long-term mortality, measured at 18–24 months, associated with previous transfusion complications/events in the neonatal period.

3. Adverse neurodevelopmental outcomes at 18–24 months, associated with previous transfusion complications/events in the neonatal period.

Composite outcomes of relevance or additional adverse events not previously identified will also be included.

### Search strategy

There will be no language restrictions, and we will attempt to translate articles in languages other than English, depending on translational services available. Literature published from 1990 onwards will be searched and studies clearly completed prior to 1990 will be excluded. These studies will be excluded as since the 1990s, increasingly restrictive RBC transfusion practices have been introduced and changes in RBC products transfused (primarily leukoreduction) have occurred. Literature and studies from 1990 onwards are more likely to reflect current neonatal transfusion practices. We will include studies available as full-text publications only as it will be difficult to apply all selection criteria and extract data for abstract-only publications. A comprehensive search of the following bibliographic databases will be performed, including MEDLINE (PubMed/OVID), EMBASE (OVID) and the CENTRAL database of the Cochrane Library. We will also undertake hand searching of reference lists and contact authors of relevant studies. We will not review other grey literature. The search strategy will include only terms relating to and describing the participants and the intervention. We will use both free-text terms and controlled vocabulary.

### Selection of studies

Two reviewers will independently screen all electronically derived citations/abstracts of papers identified by the review search strategy for relevance. At this stage, screening will be based on title and abstract, and only clearly irrelevant studies will be excluded. Full text will be obtained for a selection of potentially relevant studies. The two reviewers will then formally assess the full texts for eligibility. If necessary, further information will be sought from the authors where articles contain insufficient data to make a decision about eligibility. Potential disagreements between the review authors will be resolved by consensus. If an agreement cannot be reached, a third reviewer will adjudicate. Details of excluded studies will be recorded as well as reasons for exclusion. The review authors will not be blinded to names of authors, institutions, journals or the outcomes of the trials. If any of the review group is an author on a paper identified in the search, they will be excluded from making a decision whether or not to include the study in the review, and another member of the group will make the decision.

### Data extraction

Two authors will conduct data extraction independently using a data extraction form designed and piloted specifically for this systematic review. The pilot process for the data form will involve the two authors extracting data from at least one of each of the included study types for the review. The data extraction forms will then be reviewed by the two senior members of the authorship group and revised as required. Data extracted will include information regarding study design, participants, definitions of adverse effects and associations (outcomes), RBC transfusion regimen and the control/comparison if applicable, neonatal adverse effects reported and results relevant to the review, the risk of bias assessment, including an assessment on confounding, relevance and funding sources. Specific details regarding adverse effects and associations, including grade or severity, will also be collected including were they clearly defined *a priori* and what was the period of follow-up of study participants.

If an agreement cannot be reached over any aspects of data extraction, a third reviewer will adjudicate.

## Methodological quality assessment and risk of bias assessment

Studies will not be excluded based on quality of research methods. A formal risk of bias assessment will be performed. For randomised controlled trials, the Cochrane Collaboration's tool for assessing risk of bias will be used. For non-randomised studies, a modified Newcastle-Ottawa Scale (NOS) will be used to assess the quality of non-randomised studies and it will also be used to assess those without a comparator group. We are aware that the Cochrane Collaboration is developing a new risk of bias tool for non-randomised studies. If a working draft is available in time, we will also consider relevant items from this tool for inclusion into our risk of bias assessment (modified NOS). We plan to undertake sensitivity analysis by grading studies at low or high risk of bias (qualitative assessment only). We will factor in all aspects of risk of bias, for both qualitative and quantitative syntheses, when interpreting the evidence, and this will include formal risk of bias assessments, study design and quantity of data. We will separately present findings in tables for comparative and non-comparative studies. Although conclusions will be drawn from both groups, the focus of interpretation will be on studies with comparator arms, and this will apply for any quantitative analysis.

## Analysis plan

### Qualitative synthesis

The main analysis will be descriptive. We will provide a qualitative synthesis from the eligible studies, categorised by the type of adverse effect for primary outcomes and causal pathway for secondary outcomes. This section aims to provide a summary of adverse effects attributed to the receipt of RBC transfusion in the neonatal population.

### Quantitative synthesis

If data allows a quantitative analysis of outcome data, we will analyse separately randomised and non-randomised studies. We are expecting that there will be heterogeneity among included studies, and hence, random effects models will be used to calculate separate pooled estimates for each study type. If available and according to study design, odds ratios (ORs), risk ratios (RRs), hazard ratios (HRs) and incidence ratios (IRs) will be pooled separately. If the number of studies providing data is small, and if the number of events is rather small, then it is expected that these relative measures will yield similar results. In this case, and in order to reduce heterogeneity and provide more robust estimates, we will attempt to transform ORs, RRs and HRs into a single metric [22], and we will support this strategy with a sensitivity analysis by type of measure.

We will explore clinical heterogeneity concentrating on the different RBC transfusion strategies and settings. Statistical heterogeneity (where meta-analysis is feasible) will be assessed by the *I*^2^ test, with values above 80% classed as considerable heterogeneity. We will approach pooling cautiously, and if *I*^2^ > 80%, we will not provide pooled results, but instead we will provide information either on a table or an un-pooled forest plot. If the data permits, we will carry out subgroup analysis and sensitivity analysis based on the different types of effect measure (if they have been combined as mentioned earlier). We will also carry out sensitivity analysis based on the risk of bias assessment in terms of selection bias, and any identified confounding factors.

## Discussion

This systematic review will identify and synthesise the reported adverse effects and associations of RBC transfusions in the neonatal population.

The limited reporting of adverse effects in neonatal transfusion trials, the quality of the studies identified as well as the risk of bias inherent in studies in this area are likely to be significant limitations to our review [9]. However, the identification and collation of all current known adverse effects due to, or associated with, RBC transfusion in neonates are key steps in improving the reporting of these important events. The need for standardised neonatal definitions for all relevant adverse effects is also likely to be highlighted by this review, as well as the need for consistent reporting.

By drawing together the current known adverse effects and associations of RBC transfusion in neonates, we aim to provide a clear overview of this area and clarify future research areas. This protocol may also be used in the future to examine the potential adverse effects of other blood products and intravenous fluids used in the neonatal population. We believe that this systematic review is timely and will make a valuable contribution through highlighting existing research gaps.

## Abbreviations

EMBASE: Excerpta Medica Database; OR: Odds ratio; PRISMA: Preferred Reporting Items for Systematic Reviews and Meta-Analyses; PROSPERO: International Prospective Register of Systematic Reviews; RBC: Red blood cell; RR: Risk ratio.

## Competing interests

The authors have no competing interests to declare.

## Authors’ contributions

AK, SP, MT, LL, JC, NS and SS participated in the design of the protocol and helped to draft the manuscript. All authors read and approved the final manuscript.

## Authors’ information

AK is a consultant neonatologist at the Women's and Children's Hospital, Adelaide, and a higher-research-degree student at the University of Adelaide, South Australia, Australia. SP is a neonatal research fellow at Cambridge University NHS Foundation Trust, Cambridge, UK. MT is a senior medical statistician (systematic review methodology) at the Centre for Statistics in Medicine, University of Oxford. She is also a training co-ordinator for the Cochrane Collaboration, Oxford, UK. LL is a paediatric haematologist and transfusion medicine specialist at Sunnybrook Health Sciences Centre and the University Health Network in Toronto, Canada. JC is a haematologist and transfusion medicine specialist at Sunnybrook Health Sciences Centre and the University Health Network in Toronto, Canada. NS is a haematologist and transfusion medicine specialist at Mount Sinai Hospital in Toronto, Canada. SS is a consultant haematologist for NHS Blood and Transplant at the John Radcliffe Hospital, Oxford, UK. He is an honorary consultant paediatric haematologist at Oxford University Hospitals NHS Trust and honorary senior clinical lecturer at the University of Oxford.
